# Super Resolution of Magnetic Resonance Images

**DOI:** 10.3390/jimaging7060101

**Published:** 2021-06-21

**Authors:** Prabhjot Kaur, Anil Kumar Sao, Chirag Kamal Ahuja

**Affiliations:** 1 Indian Institute of Technology Mandi, Mandi, Himachal Pradesh 175005, India; anil@iitmandi.ac.in; 2 Post Graduate Institute of Medical Education & Research, Chandigarh 160012, India; chiragkahuja@rediffmail.com

**Keywords:** reconstruction, super resolution, enhancement, self-similarity, MRI

## Abstract

In this work, novel denoising and super resolution (SR) approaches for magnetic resonance (MR) images are addressed, and are integrated in a unified framework, which do not require example low resolution (LR)/high resolution (HR)/cross-modality/noise-free images and prior information of noise–noise variance. The proposed method categorizes the patches as either smooth or textured and then denoises them by deploying different denoising strategies for efficient denoising. The denoising algorithm is integrated into the SR approach, which uses a gradient profile-based constraint in a sparse representation-based framework to improve the resolution of MR images with reduced smearing of image details. This constraint regularizes the estimation of HR images such that the estimated HR image has gradient profiles similar to the gradient profiles of the original HR image. For this, the gradient profile sharpness (GPS) values of an unknown HR image are estimated using an approximated piece-wise linear relation among GPS values of LR and upsampled LR images. The experiments are performed on three different publicly available datasets. The proposed SR approach outperforms the existing unsupervised SR approach addressed for real MR images that exploits low rank and total variation (LRTV) regularization, by an average peak signal to noise ratio (PSNR) of 0.73 dB and 0.38 dB for upsampling factors 2 and 3, respectively. For the super resolution of noisy real MR images (degraded with 2% noise), the proposed approach outperforms the LRTV approach by an average PSNR of 0.54 dB and 0.46 dB for upsampling factors 2 and 3, respectively. The qualitative analysis is shown for real MR images from healthy subjects and subjects with Alzheimer’s disease and structural deformity, i.e., cavernoma.

## 1. Introduction

High quality magnetic resonance (MR) images are generally desired for precise medical diagnosis and analysis, and are typically characterized by high spatial resolution and high signal to noise ratio (SNR). However, the acquisition of such high quality MR images is limited due to various constraints such as acquisition time and requirement of expensive hardware. For example, an increase in the spatial resolution is limited as it leads to a decrease in SNR in MR images for a fixed acquisition time. It has been experimentally demonstrated that the post-acquisition MR image processing can increase the spatial resolution of MR images along with improved SNR [1,2]. The focus of this work is to improve the resolution (using a super resolution (SR) approach) and SNR (using a denoising approach) of MR images.

*Different paradigms of machine learning approaches:* The limited availability of example/training MR images makes it crucial to classify the existing denoising and SR approaches based on the requirement of paired/labeled example data as (i) supervised and (ii) unsupervised. The supervised approaches require paired example images for learning the mapping between input and ground truth images. The unsupervised approaches, on the other hand, are not supervised by the paired/labeled data. Several unsupervised approaches have been addressed in the literature which require unpaired/unlabeled data to learn the mapping between input and ground truth images. For example, the cycle-generative adversarial networks (GAN)-based unsupervised SR approach exploits transductive learning and requires example low resolution (LR) and high resolution (HR) images without any correspondences among them [3]. Many existing unsupervised SR approaches require either example LR or example HR or example cross-modality MR images [4,5,6,7,8,9,10,11]. Another class of unsupervised approaches assumes a mathematical model between input and desired output images, and poses the problem as ill-posed inverse problems [12,13,14,15,16,17]. The main advantage of these approaches is that these approaches do not require any example images. However, there might be other requirements in these approaches such as several denoising approaches from this class which obviate the requirement of example images and require the prior knowledge of noise: noise variance or distribution of noise [14,15,16,17]. The denoising approaches which exploit prior knowledge of noise have been observed to outperform the approaches which do not require the prior knowledge of noise, but such requirements of noise are generally not met because the variance of noise is often not known a priori. Several hybrid versions for supervised and unsupervised frameworks also exist such as a semi-supervised framework which requires a few paired and many unpaired example MR images, for example cycle GAN-based methods [18].

In the last decades, several deep learning-based approaches have been addressed to super resolve the MR images [7,19,20]. These methods use three dimensional convolutional neural networks in [7] to learn the mapping among given LR and HR images to estimate HR images. Such methods are further investigated to estimate an HR image along with learning the residual/difference in estimated HR and ground truth images to improve the estimation of HR images [19]. A similar residual learning-based strategy is used for super resolving the musculoskeletal-knee MR images in the slice-select direction [20]. Most of the addressed deep learning-based methods are supervised and the recent research is inclined towards reducing the requirement of example images [18].

The performances of supervised, semi-supervised, and unsupervised approaches that exploit example images are better than the latter class of unsupervised approaches without the requirement of any example images, but the applicability of these approaches is highly restricted due to the limited availability of example images in real scenarios [3,18]. Further, the performance of supervised approaches is highly dependent on the training database [21]. These issues can be addressed by unsupervised approaches, which obviate all requirements of example data, but there are very few in the literature [12,13]. Therefore, in this direction, we focus on SR and denoising approaches in an unsupervised framework, which obviates the additional requirements such as prior-knowledge or estimation of noise variance and the distribution of noise, along with the requirement of example images. For simplicity, the latter class of unsupervised approaches, which do not require any example images, are here onwards referred to as unsupervised approaches.

*Challenges in unsupervised image denoising:* Image denoising specifically for MR images is well explored using Bayesian approaches [22], partial differential equation [23], adaptive smoothing and filtering [24], collaborative filtering [25,26,27], etc. The low-rank approximation (LRA) of a given image patch estimated by either a nuclear norm or singular value decomposition (SVD) or principal component analysis (PCA) has been extensively studied for denoising of MR images [28,29,30,31]. The LRA-based image denoising methods, which use PCA to provide the first few (*l*) eigenvectors with significant eigenvalues that are used to represent denoised image patches, can be categorized as unsupervised approaches. The performance of these methods depends upon the criterion to choose the appropriate *l* to discard l+1 onward eigenvectors, which are mainly associated with noise present in the given image patches. Using the same criterion (e.g., the same *l*) to select the eigenvectors for denoising different kinds of image patches (such as smooth patches, and patches with varying strengths of edges) may not be suitable to improve the trade-off between suppression of noise and smearing of edges in images. The issue of the same criterion to choose *l* for different patch structures has been addressed by choosing *l* adaptive to the patch structure as in [14,15,16,17]. These approaches address the computation of weights that are adaptive to the patch structure. These weights are used to weigh the projection coefficients of image patches on eigenvectors to reconstruct the denoised image patches. Hence, it leads to efficient denoising of an image. For smooth/flat image patches, the approaches in [14,17] provide exactly zero weight, and when multiplied with projection coefficients (producing l=0), they lead to better denoising of smooth patches. However, these approaches require prior knowledge of variance of noise for the computation of adaptive weights. Other approaches [15,16] do not require prior knowledge of noise but in these approaches, the value computed for *l* is usually greater than or equal to one even for smooth patches and thus may result in noisy smooth patches. This concern is addressed by the proposed denoising approach in the unsupervised framework by categorizing the smooth and textured patches without the requirements of prior knowledge of noise variance and any example image. The categorized patches are denoised using different denoising strategies. This leads to effective denoising of smooth patches similar to [14,17] and better than [15,16] but without the requirement of noise variance in the proposed denoising approach.

*Challenges in unsupervised SR:* The initial attempt made in unsupervised SR of MR images is based on the non-local means (NLM3D) [13]. In this approach, each small 3D cube volume is represented using weighted averaging of similar 3D cubes present non-locally in interpolated MR image volume. Here, the weighted averaging of image patches generally tends to blur the image details. Though weighted averaging aids in its robustness to noise while super-resolving the noisy images, it can be effective for only a small strength of noise. Feng Shi et al. exploit the low-rank structure of brain MR image patches along with the total variation regularization in MR images low rank and total variation (LRTV) [12]. This approach reduces the blur in reconstructed image details but is observed to have a tendency to produce staircase artifacts in reconstructed MR images (a staircase artifact is generally associated with TV prior [32]). On the other hand, the degradation of gradient profiles in MR images differs from natural images. Thus, such a difference shall be considered while deploying the gradient-based prior in the SR of MR images. The proposed SR approach addresses this difference and utilizes it to regularize the estimation of HR images such that the gradient profiles of an HR image (depicted by tissue boundaries) are restored with better accuracy and without the significant generation of artifacts like the staircase artifact.

*Scope of this work:* This paper addresses the SR of noisy brain MR images by proposing novel unsupervised denoising and a super resolution approach, which are embedded in a unified framework. The proposed denoising approach is based on the hypothesis that the trade-off between suppression of noise and smearing of image details can be improved if different structures of patches are denoised with different strategies. The categorization of different patch structures, such as smooth patches and textured/edge patches, from noisy image patches is a challenging task, especially without the prior knowledge of noise variance. The proposed work addresses such patch categorization by exploiting the relation between different structures of MR image patches and their respective low rank approximation (LRA)-based reconstruction error. The LRA-based reconstruction error of a patch here denotes the mean square error between the patch and its low-rank approximated version. Further, we propose to use a statistical measure of LRA-based reconstruction error values of an ensemble of image patches to indicate the strength of noise, and is used to categorize the patch structures adaptive to the noise strength. The eigenvectors used to compute LRA of the image patches are estimated from the ensemble of given noisy image patches. Thus, the estimated low-rank structure of image patches possesses inaccuracy. To address this, we progressively reduce such inaccuracy by re-estimating the LRA of patches from the denoised image patches in an iterative manner. This aids in the accurate reconstruction of image details. On the other hand, the gradient profiles of image details convey important semantic information and are generally degraded while improving the resolution. The gradient information thus shall be preserved to aid the reconstruction of clear tissue boundaries in MR images. To do so, we propose to regularize the estimation of the HR image in the SR approach with approximated reference gradient profile sharpness (GPS) values. The unavailable reference GPS values, i.e., GPS values of an HR image, are approximated by estimating a piecewise linear relation (for natural images the relation is linear) between GPS values of available LR and upsampled LR images. The super resolution approach addressed in this work exploits the sparse representation framework and uses the self-similarity concept to utilize similarity among patches of the test image itself for enriching the dictionary construction.

The novelty of this work is as follows: (i) a novel denoising approach is proposed that addresses the categorization of noisy image patches as smooth or textured/edges without the estimated/prior knowledge of noise such as noise variance, and (ii) a novel gradient profile sharpness-based constraint is proposed specific to the structure of MR images in the super resolution approach.

The preliminary work by us for in-plane (2D) super resolution of MR images with GPS constraint has been reported in [33]. The work in this paper has been further extended by defining a novel denoising approach that addresses the inhomogeneity among image patches and formulates a noise adaptive approach for categorization of different patch structures and for improving the resolution of noisy three dimensional (3D) MR volume. The extensive experimental analysis is reported in this manuscript including the analysis of the parameters in the proposed approach, and the performance on both healthy and unhealthy subjects.

*Contributions of our work:* The important contributory aspects of the proposed work are as follows: (i) The relation between structure of a patch and its LRA version is exploited to categorize the patch structure without the requirements of prior information about noise, (ii) the statistical mean of LRA-based error values of patches is used to indicate the strength of noise and is used to categorize the patches adaptive to the strength of noise, (iii) a progressive improvement in the estimated low-rank structure of image patches is addressed by re-estimating the eigenvectors from denoised image patches for better reconstruction of image details, (iv) the piecewise linear relation among GPS values of LR and an upsampled LR image is formulated to regularize the estimation of a denoised HR image that aids in estimation of clearer tissue boundaries, (v) extensive experimental analysis is performed for healthy and unhealthy subjects to validate the accurate estimation of MR image details.

The organization of the paper is as follows: Section 2 is an overview of related SR and denoising approaches. The proposed denoising approach and SR approach are discussed in Section 3. The experimental results are demonstrated and discussed in Section 4. The work is summarized in Section 5.

## 2. Related Works

The SR of noisy MR images has been addressed in the literature in two ways: (i) using a disjoint combination of SR and denoising approaches (first denoise and then super resolve the images or vice-versa) [12,13,25,34,35], and (ii) using the embedded framework for SR and denoising approaches [21,36]. It has been demonstrated that the image details are restored with better accuracy in the case of the embedded framework than the disjoint combination [21,36]. The existing works to improve the quality of MR images are explained in three categories: approaches that address (i) both SR and denoising in a single framework, (ii) denoising only, and (iii) SR only, in the following Section 2.1, Section 2.2 and Section 2.3, respectively. Here, we only discuss the closely related approaches in detail.

### 2.1. Embedded Approaches for SR and Denoising

The embedded frameworks integrate the denoising approach in the SR approach using the framework of either sparse representation of image patches [15,21,36,37] or neural networks, and thus omit the drawbacks of the disjoint combination of SR and denoising approaches. Numerous ways to shrink the coefficients in the wavelet domain [24] and PCA [15] have been reported in the literature to denoise while super resolving the images, but require prior knowledge of noise variance. In the case of medical images, especially for MR images, D. Trinh et al. proposed a single framework for SR and denoising [21]. Here, each HR image patch is estimated from the linear combination of a few selected example HR image patches, for the given LR image patch. However, this method requires example HR images and is thus supervised in nature with limited applicability in the real scenario. The approach in [36] addressed a sparse-representation framework for SR of noisy MR images without the requirement of example images. However, it follows the denoising approach as in [14] and thus requires prior knowledge of the strength of noise, i.e., noise variance.

### 2.2. Image Denoising Approaches

In magnitude MR images (such as T1 and T2 weighted MR images), the noise follows a Rician distribution [38]. However, for high SNR regions (pixels/patches with brain tissues of high signal intensities) the distribution of noise can be well approximated by a white Gaussian distribution [38,39]. Hence, the denoising of MR images for both Gaussian distributed noise (for high SNR regions) [28,29,40,41] and Rician distributed noise along with mean removal [25,42] is addressed in the literature. Further, many image denoising techniques in the MR images’ literature process the 3D image’s volume [27,28,35,41]. That means while denoising one MR image, the details from its adjacent slices are also considered. Apart from 3D level processing, there exist MR image denoising methods which process each image independently (2D) and do not consider the information from other slices in the same volume [34]. In LRA-based unsupervised denoising approaches, the low rank structure estimated by eigenvectors possess inaccuracy. This is because the eigenvectors, employed to reconstruct the denoised image, are estimated from the given noisy image patches (especially in unsupervised framework) and thus may not represent the reliable low-rank structure of image patches. This is addressed by an LRA-based denoising approach which is closely related to our method, and is addressed in computer vision literature, i.e., local patch group-based PCA [14]. In this method, similar patches are grouped and eigenvectors are estimated from the covariance matrix of patches in each cluster. The eigenvectors are re-estimated from the denoised images to improve the accuracy of estimated low rank structure of image patches. The projection coefficients for a given patch on the eigenvectors are adaptively weighted according to the structure of the patch for efficient denoising. However, this approach requires the prior knowledge of noise variance for the computation of these adaptive weights, in contrast to the proposed denoising approach.

### 2.3. Super Resolution Approaches

The SR approaches to improve the resolution of MR images have been well explored using different methods [4,5,6,7,9,10,12,13,43,44,45,46,47,48,49,50], and in different frameworks like sparse representation framework, deep neural networks, etc. [2,6,7,8,11,49]. Various regularizers are explored in these frameworks such as sparse derivative priors [50], gradient [49], and structural priors [47] for better restoration of HR image details. The low rank and total variation priors used in [12] aid in reconstruction of sharp image details but have been observed to induce the staircase effect [32]. The SR approach is closely related to the proposed SR approach [51] in that it addresses GPS-based approximation of the gradient field for natural images and uses the approximated gradient field to constrain the estimation of the HR image. The relation between GPS values of LR and upsampled LR natural images is approximated by linear relation and is used to estimate GPS values of the HR image. However, this approach addresses the GPS values obtained using fixed shape edge models like the triangle and mixed Gaussian edge models. These models may not fit the edges/gradient profiles of MR images. Thus, the proposed work utilizes the similar formulation of GPS from [51], but is defined using a more generalized edge model [52] for MR images instead of fixed shape edge models. Further, it has been observed that the GPS values for LR and upsampled LR MR images are not linearly related as in natural images but are piecewise linearly related [33]. We have focused on the gradient-based prior specific to MR images which addresses the above-mentioned difference between MR and natural images.

## 3. Method

For the evaluation of the proposed method, both simulated as well as real T1 weighted brain MR images are used. The simulated images are obtained from MR images dataset-I (https://brainweb.bic.mni.mcgill.ca/ accessed on 1 December 2019) and eight real MR image volumes are randomly selected from the publicly available dataset-II (https://www.humanconnectome.org/study/hcp-young-adult/data-releases accessed on on 1 December 2019). The MR images are obtained from dataset-I using a normal brain database with slice thickness 1 mm and 0% intensity non-uniformity. The selected MR images from dataset-II are acquired from a 3T MRI scanner using Magnetization Prepared Rapid Acquisition Gradient Echo (MPRAGE) sequence, time to repeat (TR) = 2400 ms, time to echo (TE) = 2.14, time inversion (TI) = 1000 ms, and flip angle (FA) = 8°. The spatial resolutions of each volume in dataset-I and dataset-II are 181×217×181 and 173×173×207, respectively, which are used to simulate noisy LR images, and then super resolved and/or denoised using different algorithms. The performance analysis is also evaluated for real MR images with uncommon image details such as structural deformity (cavernoma is found in one subject from dataset-II), and another subject with Alzheimer’s disease from dataset-III which is acquired for the Alzheimer’s Disease Neuroimaging Initiative [53]. The selected MR images in dataset-III are acquired from a 3T MRI scanner with an MPRAGE sequence with a spatial resolution of 196×256×256.

In the proposed method, an embedded framework for SR and denoising of MR images in an unsupervised framework is addressed. The proposed denoising method is built upon an LRA-based unsupervised framework for image denoising [15,29,30]. The novelty of the proposed denoising work lies in the method addressed to categorize the patch as smooth or textured, without the prior/estimated knowledge of noise variance. Further, the proposed idea to differently denoise the categorized patches leads to efficient denoising, and the re-estimation of LRA leads to better representation of image patches. This aids in improving the trade-off between noise suppression and smearing of edges. The proposed SR approach is developed upon the existing sparse representation-based framework for MR image patches [6,12]. The GPS values of brain MR images convey important semantic information such as tissue boundaries and should be preserved well and close to the gradient profiles in HR brain MR images. In the case of the proposed SR approach, the novelty lies in formulating a piecewise relation between GPS values of LR and upsampled LR images to estimate GPS values of unknown HR images. The estimated GPS values are used to regularize the estimation of HR images.

**Figure 1:**
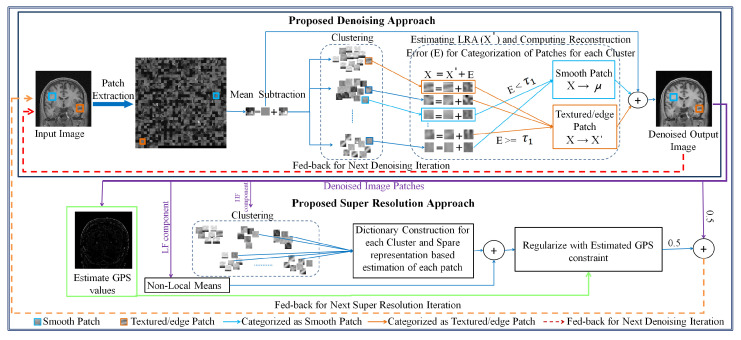
Detailed illustration of the proposed approach.

The initial HR image estimate is approximated by interpolating the noisy LR image which is denoised using the proposed denoising approach. Each iteration of the proposed SR approach is integrated with the proposed denoising approach, consisting of three consecutive steps in each iteration: (i) estimate the HR image by estimating its high-frequency component in a sparse representation framework and estimate its low-frequency component using a non-local means approach, (ii) apply the GPS-based regularization to enhance the image edge details, i.e., tissue boundaries, and (iii) the regularized image is denoised using the proposed denoising method to suppress the leftover noise. The SR approach provides better restoration of fine image details and the denoised image patches possess improved SNR, and are thus averaged for the next iteration of the SR algorithm. These three steps are followed by back projection and are repeated until the changes in the estimated HR image are insignificant. The flow of these steps in the proposed SR approach for noisy images and detailed steps for both SR and denoising are illustrated in Figure 1. The details of the proposed denoising and SR approaches are given in Section 3.1 and Section 3.2, respectively.

### 3.1. Proposed Denoising Method

The MR brain image consists of different kinds of patches such as patches with edges/texture depicting tissue boundaries, referred to as signal enriched (SE) patches (high signal related information) and patches with the relatively smooth region due to white matter and gray matter, referred to as noise enriched (NE) patches in the case of presence of noise.

It has been conjectured that noise belongs to the high-frequency range of the image spectrum, so the high-frequency (HF) component of an image patch is more contaminated with noise as compared to its low-frequency (LF) component. Thus, the HF component ($x_{h}$) of a noisy image patch is processed for denoising and the respective LF component is added later in the estimated denoised HF component of image patch. The decomposition of LF ($x_{l}$) and HF ($x_{h}$) components of an image patch (x) is performed as $x=x_{h}+x_{l}$, where $x_{l}$ is the vector with each element as $\mu =\frac{\sum_{i=1}^{m}x\left[i\right]}{m}$. Here, x=[x[i]],i=1tom, and *m* represents the dimensionality of a patch vector.

In the proposed denoising method, the HF components of patches are extracted and grouped into clusters using the Euclidean distance-based k-means algorithm (for simplicity, we will omit the notion of an HF component of the patch from here onward). The grouping of patches helps in improving the homogeneity among patches in a cluster. However, the clustering may not be accurate due to the presence of noise, thus leading to certain inhomogeneity left in the clusters. We categorize the image patches in each cluster further into smooth/NE and textured/edge/SE patches using the LRA-based reconstruction error of a patch. The LRA of a patch $x_{h}$ is obtained as ${\hat{x}}_{h}=\sum_{i=1}^{l}c_{i}u_{i},l<m$. The LRA-based reconstruction error for the *j*th patch is represented by $e_{j}$ and computed as

(1) $$e_{j}=\left|\right|x_{h}^{j}-{\hat{x}}_{h}^{j}{\left|\right|}_{2}^{2}\leq \sum_{i=l+1}^{m}\left|\right|c_{i}{\left|\right|}^{2}$$

where $x_{h}^{j}$ represents the HF component of the *j*th patch, ${\hat{x}}_{h}^{j}$ represents the LRA of the *j*th image patch, and the respective projection coefficient of $x_{h}^{j}$ on *i*th eigenvector is represented by $c_{i}$. The *i*th unit norm eigenvector is represented by $u_{i}$, after arranging eigenvectors according to descending order of eigenvalues. The inequality in Equation (1) holds true in general by keeping the eigenvectors of unit norm [54]. The LRA-based reconstruction error of a patch is upper bounded by its projection coefficients on the last eigenvectors. The different behavior of projection coefficients for different patch structures is used to categorize the patches. The details for categorization of the patch structure for each cluster and the denoising strategies for different patch structures and re-estimation of eigenvectors are given in Section 3.1.1 and Section 3.1.2.

#### 3.1.1. Categorization of Smooth and Textured/Edge Patches

It has been observed that the smooth (NE) patches have smaller magnitude variations (due to noise) than the textured/edge (SE) patches (due to edges of varying strengths). Thus, the projection coefficients ($c_{i}$) in smooth patches are constrained to small values in contrast to textured/edge patches. Following this and from Equation (1), small projection coefficients in smooth patches lead to less LRA-based reconstruction error ($e_{j}$) for smooth patches than textured/edge patches, and thus can be used to categorize the patch as smooth or textured/edge.

**Figure 2:**
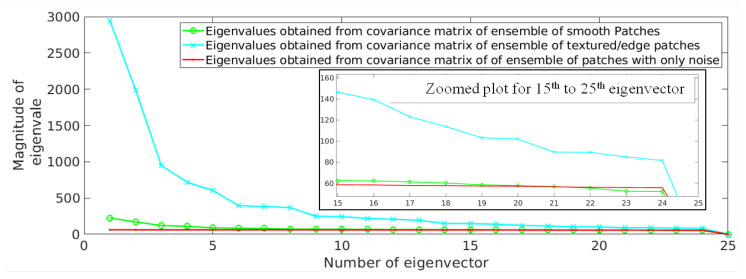
Demonstration of eigenvalues obtained from an ensemble of smooth patches (green) and textured/edge patches (cyan) present in a randomly selected magnetic resonance (MR) image degraded with 2% Rician distributed noise. The eigenvalues for patches with only noise are shown in red color. The x-axis denotes the number of eigenvector and the y-axis denotes the corresponding eigenvalue. The plot is zoomed for the 15th to the 25th eigenvectors and is shown to better visualize the small differences among eigenvalues. Here, 2% noise denotes that noise variance is 0.02×255=5.1 for an image with 255 as its maximum pixel intensity.

This has been experimentally demonstrated in Figure 2 by computing the eigenvalues for textured/edge and smooth patches, separately. The patches extracted from a randomly selected noisy MR image (degraded with 2% noise) are categorized as smooth and textured/edge. A threshold is defined to divide the reconstruction error values into two ranges, each corresponding to either smooth or textured/edge patches and is discussed in the following subsection. We have considered patches with less reconstruction error ($e_{j}$) as smooth patches and other patches as textured/edge patches, i.e., patches with varying strength of edges. The eigenvalues computed for smooth and textured/edge patches are shown using green and cyan color, respectively, in Figure 2. It can be observed that the textured/edge patches tend to obey the power law, i.e., the eigenvalues decay drastically from the first eigenvector to last eigenvector. However, the last eigenvalues of textured/edge patches are still higher than the eigenvalues of smooth patches, as shown in Figure 2, leading to higher reconstruction error for textured patches than smooth patches (followed from Equation (1)).

**Estimation of threshold:**

It shall be noted that the choice of threshold for reconstruction error of a patch ($e_{j}$) to categorize the patch as smooth or textured is crucial, and is challenging in the presence of noise. Since LRA-based reconstruction error of a patch changes with the strength of noise, the threshold should also be adaptive to the strength of noise.

**Figure 3:**
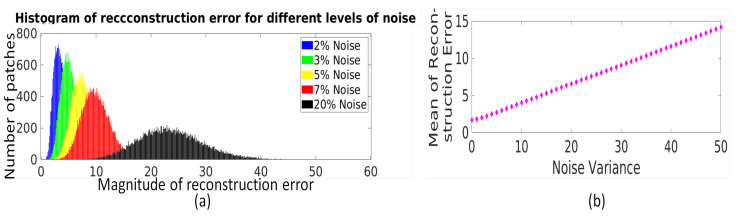
(**a**) Illustration of the relation between distribution of LRA-based reconstruction error values (e) of an ensemble of image patches and strength of noise, i.e., noise variance. (**b**) Illustration of the relation between mean of reconstruction error values with strength of noise, i.e., noise variance.

*Adaptation of threshold to the strength of noise in image patches:* The LRA-based reconstruction error for each patch (represented by $e_{j}$ for the *j*th patch) is concatenated to form reconstruction error vector for ensemble of patches as $e=[e_{1}e_{2}e_{3}\ldots e_{N}]$. The LRA-based reconstruction error for an ensemble of patches, i.e., e, is used to characterize the strength of noise present in image patches. For fixed *l*, an increase in noise variance leads to an increase in each eigenvalue and thus an increase in the reconstruction error of each patch. To demonstrate this, a randomly selected MR image is synthetically corrupted with Rician noise with different noise variance values and the histogram of reconstruction error values of all image patches (e) for different noise variance values is shown in Figure 3a. It can be observed that the histogram of reconstruction error values shifts right with increase in noise variance. The same has been observed for image corrupted with the Gaussian noise. Hence, a statistical measure of reconstruction error values obtained for image patches like mean of e computed as $\kappa =\sum_{j=1}^{N}\frac{e_{j}}{N}$, can be used to correlate with strength of noise. Here, *N* represents the number of patches. The experimental demonstration of relation between mean of reconstruction error values (κ) and noise variance is shown in Figure 3b, and it can be observed that κ follows the linear relation with noise variance.

Hence, the adaptation of the threshold to strength of noise can be taken care of by choosing the threshold which is linearly related with κ, mean of reconstruction error (from Figure 3b). Following this, the categorization of an image patch ($x_{h}^{j}$) is done as

(2) $$\mathrm{patch}\;\mathrm{kind}=\left\{\begin{array}{cc} \mathrm{textured}/\mathrm{edges}, & if\;\tau_{1}<e_{j}<\tau_{2}\\ \mathrm{smooth}, & otherwise. \end{array}\right.$$

Here, $\tau_{1}=\zeta_{1}\sum_{j=1}^{n^{{}^{\prime }}}$$\frac{e_{j}}{n^{{}^{\prime }}}$, and $\tau_{2}=\zeta_{2}\sum_{j=1}^{n^{{}^{\prime }}}$$\frac{e_{j}}{n^{{}^{\prime }}}$ such that $\zeta_{2}>\zeta_{1}$. The values for $\zeta_{1}$ and $\zeta_{2}$ are empirically chosen, and are described in the experimental section. The $e_{j}=\left|\right|x_{h}^{j}-{\hat{x}}_{h}^{j}{\left|\right|}_{2}^{2}$ represents reconstruction error of the *j*th patch, $x_{h}^{j}$ represents the HF component of the *j*th patch and $n^{{}^{\prime }}$ represents the number of patches in the selected cluster. Here, $\tau_{1}$ and $\tau_{1}$ represent the noise adaptive thresholds used to categorize the image patch structure. The $\tau_{2}$ represents the threshold for patches having higher projection coefficients only along the last few eigen directions. Such patches are usually rare but should also be denoised in the same manner as smooth patches. This is because the reconstruction of a patch using only the last few eigen directions conveys very little semantic meaning about image details and is usually considered to possess noise like characteristics.

**Figure 4:**
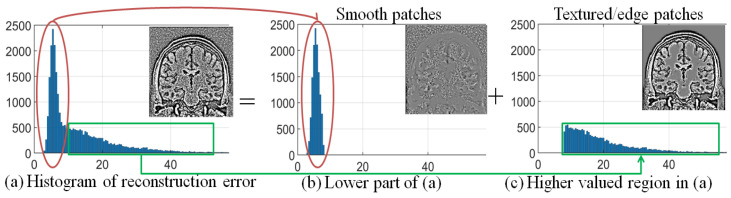
Demonstration of the relation between reconstruction error vector e and patch structure, i.e., smooth and textured/edge, using the noise adaptive threshold $\tau_{1}$, for (mean subtracted patches of) a randomly selected image corrupted with 2% Rician distributed noise: (**a**) Histogram of reconstruction error values e and the corresponding image, (**b**) histogram of reconstruction error values less than $\tau_{1}$ and the corresponding patches in the image, and (**c**) histogram of reconstruction error values greater or equal to $\tau_{1}$ and the corresponding patches in the image.

The reason for employing Equation (2) in the categorization of image patches is experimentally illustrated in Figure 4. It shows the histogram of reconstruction error values obtained using LRA of smooth and textured patches of a randomly selected MR image in Figure 4a. The reconstruction error values (the x-axis of Figure 4a) are split into low and high values by using a threshold ($\tau_{1}$), defined using the mean of the reconstruction error of the ensemble of patches and considering $\zeta_{1}=0.7$, i.e., $\tau_{1}=0.7\sum_{j=1}^{n^{{}^{\prime }}}$$\frac{e_{j}}{n^{{}^{\prime }}}$. The patches corresponding to low ($<\tau_{1}$) reconstruction error values are shown in the red box, and patches corresponding to high ($\geq \tau_{1}$) reconstruction error values are shown in the green box. These patches are placed on their corresponding locations in 2D to form an image as shown in Figure 4b,c, respectively. It can be observed that the patches corresponding to low reconstruction error values are mostly smooth patches corrupted with noise, and high reconstruction error values correspond to patches dominated with texture/edges.

*Different denoising strategies for different kinds of patches:* The LRA for both smooth and textured/edge patches is computed using the considerate number of eigenvectors such that the edges are present in textured/edge patches even though the noise in these patches is not suppressed completely. The denoised version of textured/edge patches is represented by their estimated LRA, i.e., ${\hat{x}}_{h}$. The pixels in each categorized smooth patch, are replaced by the average of pixel values in that patch. The averaging acts as a low pass filter and thus denoises the smooth patches. As these are smooth patches, averaging does not lead to any significant loss of image details, thus leading to efficient denoising.

#### 3.1.2. Re-Estimation of LRA in an Iterative Manner

**Figure 5:**
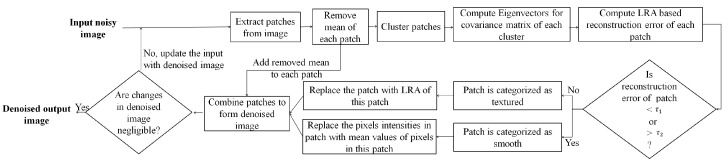
Illustration of major steps in the proposed denoising approach.

Eigenvectors play an essential role in estimation of textured/edge patches. Since the estimation of eigenvectors is done from noisy image patches, the approximation of a low-rank structure possessed by textured/edge patches may be noisy. To improve the reliability of the low-rank structure estimated by eigenvectors and for better reconstruction of image details, the proposed denoising procedure is repeated in an iterative manner. The eigenvectors are estimated in each iteration from the denoised patches obtained in the previous iteration. The patches, categorized as smooth patches in the previous iteration and denoised by an averaging operator, lie near the origin, i.e., are with insignificant values after mean removal (for extracting HF component). Thus, these patches do not play any role in the computation of eigenvectors in the next iteration. However, the averaging of categorized smooth patches in the previous iteration affects the overall distribution of patches, which leads to a change in estimation of eigenvectors in the next iteration such that the effect of noise on the computation of eigenvectors is reduced. Thus, repeating this process iteratively helps in increasing the reliability of estimated eigenvectors which capture the low-rank structure of noiseless patches, thus aiding in better reconstruction of image details. The summarized flow of steps followed in the proposed denoising approach is shown in Figure 5.

### 3.2. Super Resolution of Noisy MR Images

The above-mentioned denoising procedure of image patches is performed in each iteration of the SR algorithm (refer to Figure 1), which is based on the framework of sparse representation of image patches. In the SR approach, the LF components of HR image patches are reconstructed using a non-local means approach. On the contrary, the HF components of HR image patches are estimated using the framework of sparse representation framework. The unavailability of example HR MR images for forming the dictionary for sparse representation is addressed using the concept of self-similarity. The image is up and down scaled to exploit the self-similarity among patches of the image by forming an image pyramid. The HF components of image patches and patches from the up-down scaled version of the same image are clustered using the Euclidean distance-based k-means algorithm. The patches of an image and the up-down scaled version of the image aid in enrichment of dictionary construction. The k-means approach is a typical unsupervised method for clustering, and clusters the input image patches without any requirement of example data [54]. For each cluster, the eigenvectors are estimated from the covariance matrix of an ensemble of patches in that cluster and are concatenated column wise in the matrix, named the PCA-based dictionary (for more details about dictionary building, please refer to [33]). Please note that the estimated HR image is denoised using the proposed denoising approach in each iteration of the SR approach but the steps carried out in both are independent of each other.

The mathematical model for degradation of HR MR images for super resolution is adapted using a point spread function (H) (PSF, generally considered Gaussian kernel) and a downsampling operator (B) such as y=BHx, similar to the adaption of SR for MR images in [2,12]. Here, $y\in R^{p\times 1}$ represents the noisy LR patch vector obtained by blurring and downsampling the HR patch vector $x\in R^{q\times 1},q>p$ using $H\in R^{q\times q}$ and $B\in R^{p\times q}$, respectively. Thus, the objective function for the sparse representation-based SR algorithm with the constraint for preserving gradient profiles sharpness, proposed in this work, is as follows:

(3) $$min_{\alpha ,\eta }||y-{\mathrm{BHA}\alpha ||}_{2}^{2}+{\left||\alpha |\right|}_{1}+\lambda ||E_{\hat{x}}\left\{\eta \right\}-E_{x}\left\{\eta_{H}\right\}{\left|\right|}_{2}^{2}.$$

The interpolated version of denoised y using the proposed denoising approach is considered as the initial estimate of x. The sparse representation of an image patch is computed as $\hat{x}=A\alpha$ where A is the PCA-based compact dictionary estimated for a cluster to which the initial estimate of x belongs and α denotes the coefficient vector. Since the sparsity is implicitly induced by thresholding the insignificant projection coefficients (α), the weight for term ${\left||\alpha |\right|}_{1}$ is not mentioned. Here, λ represents the weight for the regularizer. The η and $\eta_{h}$ represent the images with GPS values for the estimated HR image (its patches are represented by $\hat{x}$) and the original HR image (its patches are represented by x), respectively. Here, the GPS values corresponding to patch $\hat{x}$ are extracted from η from the same location as patch $\hat{x}$ using operator $E_{\hat{x}}\left\{\eta \right\}$. Similarly, the $E_{x}\left\{.\right\}$ operator extracts the corresponding GPS values for patch x from ${\hat{\eta }}_{H}$. Since the reference GPS values $\eta_{H}$ are unavailable, the constraint becomes $\left|\right|E_{\hat{x}}\left\{\eta \right\}-E_{x}\left\{{\hat{\eta }}_{H}\right\}{\left|\right|}_{2}^{2}$, where ${\hat{\eta }}_{H}$ represents the approximated $\eta_{H}$. The computation of GPS values and the estimation of ${\hat{\eta }}_{H}$ are explained below.

The GPS value indicates the ratio of height (*h*) and width (*w*) of the edge centered on an edge pixel in image, i.e., h/w as defined in [51]. Higher values of *h* indicate higher contrast and high values of *w* indicate more blur in the corresponding edge. Thus, higher GPS values correspond to sharper image details. The GPS value for each pixel in an image is computed to obtain GPS valued image η. It has been observed in the case of natural images [51] that the GPS values of an HR image ($\eta_{H}$) possess a linear relation with GPS values in an upsampled LR image (UR) ($\eta_{U}$) as $\eta_{H}=\beta \eta_{U}$. However, we observed that the relations among GPS values of HR and UR MR images are not exactly linear especially when the super-resolution factor is increased (for more details, refer to [33]). It can be due to the fact that there exist many small details in MR images which are degraded severely, and due to image details with varying sharpness in MR images which are degraded differently while upscaling. Thus, there exists a non-linearity between GPS values of UR and HR MR images. This non-linear behavior is approximated with a piecewise linear relation by dividing the range of $\eta_{U}$ values into four regions. For each *i*th region of $\eta_{U}$ values, GPS values of HR can be related as

(4) $$\eta_{H_{i}}=\beta_{i}\eta_{U_{i}},\mathrm{here}\;i=1\mathrm{to}\;4.$$

The $\eta_{H_{i}}$ and $\eta_{U_{i}}$ represent the GPS values for the *i*th region of $\eta_{U}$ values. Here, $\beta_{i},\forall i=1\;\mathrm{to}\;4$, is unknown and is estimated to approximate $\eta_{H_{i}}$. It has been observed that most of the GPS values for a UR image lie in the small valued region, whereas there exist higher GPS values for both LR and HR images. Such behavior of the UR image can be due to the basic interpolation used for upsampling LR images that lead to blurred details and hence lead to small GPS values. Further, the percentage of edge pixels in LR and HR images is also generally considered as similar. Following which, the histogram of GPS values of LR images ($hist(\eta_{L})$) is assumed to be similar to $hist(\eta_{H})$. Hence, from Equation (4), we can estimate $\beta_{i}$ by minimizing the Chi-squared distance between $hist(\eta_{L_{i}})$ and $hist(\beta_{i}\eta_{U_{i}})$, for each region.

We thus estimate the GPS values for an HR image as

(5) $${\hat{\eta }}_{H_{i}}=\beta_{i}\eta_{U_{i}},i=1\;\mathrm{to}\;4.$$

using LR and UR images only. This is used to constrain the solution space for the estimate of the HR image (I) by using $\eta_{H}≃{\hat{\eta }}_{H}$ in Equation (3). Such a piecewise linear relation-based approximation for GPS values of an HR image aids in the improvement of the linear correlation of $\eta_{H}$ and $\eta_{U}$ for each region of $\eta_{U}$, thus leading to a better approximation of the underlying relation. Further, it also helps in considering the low GPS values (obtained from relatively smoother regions) and high GPS values (from regions with edges) differently, which aids better preservation of tissue boundaries. It has to be noted that the four regions for a piecewise linear relation are empirically chosen as choosing three or five regions provides lesser overall linear correlation among $\eta_{H}$ and $\eta_{U}$ values. The step wise flow of the proposed approach is shown in Algorithm 1.
**Algorithm 1** Estimate denoised HR image.**Input:** Noisy LR image patches y**Output:** Denoised HR image patches $\hat{x}$**Variables:** M: Number of iterationsk = 1 to M*Step 1:* Denoise y as discussed in Section 3.1. Interpolate the denoised LR image patches to provide initial denoised HR patches x, represented by $\hat{x}$.*Step 2:* Estimate GPS values ${\hat{\eta }}_{H}$ of $\hat{x}$ as explained in Equation (5).*Step 3:* Apply a low pass filter to $\hat{x}$ to get the LF component and subtract it from $\hat{x}$ to get the HF component of $\hat{x}$.*Step 4:* Represent the LF component of each patch of $\hat{x}$ using a non-local mean approach.*Step 5:* Apply k-means clustering to HF components of $\hat{x}$, and up-down scaled versions of image patches $\hat{x}$. Construct a PCA-based dictionary for each cluster.*Step 6:* Represent the HF component of $\hat{x}$ using one of the dictionaries, i.e., the dictionary constructed from a cluster to which the HF component of $\hat{x}$ belongs.*Step 7:* Update the estimate of the HR patch $\hat{x}$ by adding estimates of the LF (from *Step 4*) and HF (from *Step 6*) components.*Step 8:* Regularize and update the estimated HR image patch $\hat{x}$ obtained in *Step 7* using GPS constraint.*Step 9:* Denoise the estimate of HR patch $\hat{x}$ obtained in Step 8 as discussed in Section 3.1.**If**k>0 and ${\hat{I}}^{k}-{\hat{I}}^{k-1}<\epsilon$    break;**else**Update $\hat{x}$ as average of outputs from *Step 8* and *Step 9*, i.e., ${\hat{I}}^{k}$, in *Step 3* and repeat the steps from *Step 3* to *Step 9*.**end If****end while loop**

## 4. Results and Discussion

The performance of the proposed algorithm is evaluated for SR of noisy MR images as well as individually for the denoising approach and super resolution along three directions, i.e., in-plane and slice-select direction. The reconstruction quality of the resultant images is reported using peak signal to noise ratio (PSNR), structural similarity index (SSIM) [55], and feature similarity index metric (FSIM) [56] values. Higher PSNR and SSIM values indicate the overall quality improvement of the reconstructed image. Higher FSIM indicates the higher subjective evaluation based on image gradient magnitude and phase congruency, and is generally used to indicate better features related to edge information [56]. The weight for GPS regularizer (λ) is empirically chosen as 0.001 for the best reconstruction of image patches and $\zeta_{1}$ and $\zeta_{2}$ are empirically chosen as 0.7 and 1.8, respectively. The patch size used in the experiments is 5×5. The number of iterations for the denoising algorithm is fixed to 3 because it has been observed that further increase in iteration does not improve the performance. The maximum number of iterations in the proposed SR approach is fixed at 320 and is otherwise stopped early if the change in the estimated HR image is insignificant.

### 4.1. Denoising of MR Images

The experiments are performed for images corrupted with Rician and Gaussian distributed noise. The noise is synthetically added in simulated images as well as real MR images to simulate the real scenario. Here, the variance of noise is decided as a percentage of brightest tissue in an MR image, followed from the literature [34,41,57]. For example, 2% noise here means the noise variance is 0.02 × 255 = 5.1 for the image with 255 as its maximum intensity value. The proposed denoising approach is compared with conventional as well as state-of-the-art denoising methods addressed for MR images. These methods require prior knowledge of noise variance. In addition to noise variance, few approaches also require prior knowledge of noise distribution. The existing methods that process 2D images include non local means (NLM), universal NLM (UNLM) [34] and variance stabilization transform (VST) [25] with block-matching and 3D filtering (BM3D) [27]. Other existing methods denoise 3D image volume by considering the adjacent slices information. These methods include optimized blockwise NLM denoising filter optimized blockwise non local means (ONLM) [57] and its adapted version ONLM (AONLM) [40], multi-resolution-based ONLM multi-resolution based ONLM (MRONLM) [41], oracle-based discrete cosine transform filter oracle based discrete cosine transform (ODCT) explained in [57], PCA-based denoising over ODCT method PCA-based denoising over ODCT (PRINLM) [35], and VST in conjunction with block matching with 4D filtering (BM4D) and AONLM [25].

**Figure 6:**
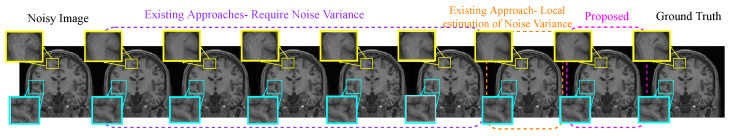
Demonstration of image denoising achieved by different denoising algorithms on real MR images with synthetically added 2% Gaussian noise. From left to right—noisy image, optimized blockwise non local means (ONLM) [57], multi-resolution based ONLM (MRONLM) [41], oracle based discrete cosine transform (ODCT), PCA-based denoising over ODCT (PRINLM) [35], variance stabilized transform with blockwise matching and 4D filtering (VST-BM4D) [25], adaptive ONLM (AONLM) [40], the proposed approach, and the original noiseless image.

One real MR image is randomly selected, degraded with 2% Gaussian noise, and is denoised using different algorithms. The denoised images are shown in Figure 6. It can be observed that the proposed approach has reduced smearing of image details (in the yellow box), provides efficient denoising in the smooth region, and estimates edges with comparable quality to existing methods in the cyan box in Figure 6.

Table 1 gives the quantitative comparison of the proposed approach with existing approaches. Here, the highest PSNR and SSIM values are highlighted in red color. PSNR and SSIM values obtained for proposed work are made bold. The blue colored values indicate the performance of existing algorithms that are comparable to the proposed work. The estimation of noise variance is crucial and is observed to be inaccurate as the level of noise increases, leading to decreased performance of the existing algorithms [58]. Hence, for a fair comparison, the existing works with the requirement of prior knowledge of noise variance are given the true variance value in the experiments.

It can be observed that the proposed work, without the requirement of knowledge of variance and distribution of noise, performs comparable to the existing classical methods such as NLM and UNLM [34], and the recent methods like ODCT and PRINLM [35] that, on contrary, have such requirements. The proposed method provides PSNR values within the 0.5 dB range of these existing approaches. This can be explained by the fact that in NLM and UNLM approaches, the images are denoised using weighted averaging and thus several image details get blurred, leading to its comparable performance to the proposed approach.

The existing supervised 3D volume-based approaches addressing denoising of MR images perform better than the proposed approach. These approaches assume the prior distribution of noise and such assumption is the reason that VST-based methods, i.e., VST+BM4D and VST+AONLM, perform better for images with Rician distributed noise than with Gaussian distributed noise. These approaches also require prior knowledge of noise variance. Hence, such prior knowledge aids in better performance for these approaches as compared to the proposed approach, but is not generally available in the real scenario.

#### 4.1.1. Significance of the Proposed Patch Categorization and Re-estimation of Eigenvectors

**Figure 7:**
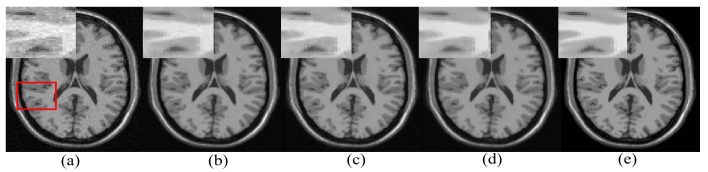
Illustration of the significance of the proposed categorization of smooth and textured/edge patches and progressive estimation of eigenvectors by adapting it with the existing low-rank approximation (LRA)-based denoised approach [15]. (**a**) Noisy image (3% Rician Noise), (**b**) denoised using the existing LRA-based denoising approach [15], (**c**) the proposed denoising approach, (**d**) the existing approach [15] adapted with the proposed patch categorization and re-estimation of eigenvectors, (**e**) noise-free image.

The significance of the proposed patch categorization is demonstrated by incorporating it in an existing LRA-based denoising method [15]. For this, the image patches are categorized as smooth and textured/edge patches using the proposed method. The smooth patches are denoised using the proposed method and the textured/edge patches are denoised using the existing method [15]. The eigenvectors in the existing method are re-estimated in the progressive manner as mentioned in the proposed denoising approach. A randomly selected denoised image (3% Rician noise) obtained by this procedure is shown in Figure 7d. The noisy input and noise-free images are shown in Figure 7a,e, respectively. The images denoised using the method described in [15] is shown in Figure 7b. It can be observed that smooth patches are not efficiently denoised in the existing LRA-based approach [15]. The denoising of such smooth patches is improvised by adapting the existing approach to the proposed denoising method indicating the advantages of patch categorization. On the other hand, the tissue boundaries represented by the proposed denoising approach are not denoised well but improvise by using patch adaptive *l* for textured/edge patches from the existing approach [15]. This can be due to the empirically chosen fixed *l* in the proposed denoising approach for representation textured/edge patches.

#### 4.1.2. Parameter Analysis for Optimal Selection of $\tau_{1}$ in the Proposed Denoising Approach

**Figure 8:**
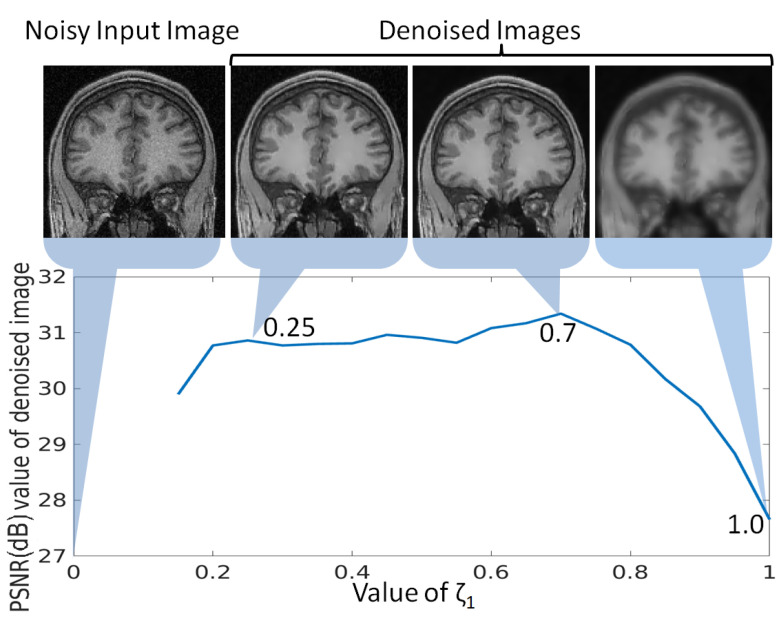
Analysis of parameter $\zeta_{1}$ for optimal selection of $\tau_{1}=\zeta_{1}\sum_{i=1}^{N}e_{i}$, $e_{i}$ denotes the low rank-based reconstruction error of the *i*th patch and *N* denotes the number of patches, for categorization of the patch as smooth or textured/edge in the proposed denoising approach. The peak signal to noise ratio (PSNR) values are computed for an MR image denoised using different values of $\zeta_{1}$, and plotted in this Figure. The denoised images using $\zeta_{1}$ as 0.25, 0.7 and 1.0 are also shown to indicate the reason for rise and drop in PSNR values.

In the proposed denoising approach, $\tau_{1}$ is a crucial parameter as it is challenging to separate the smooth and textured/edge patches in the presence of noise. In addition to the noise adaptive threshold $\tau_{1}$, the optimal threshold has to be chosen such that no texture/edge patch is categorized as smooth patch because it may lead to loss of image details. To analyze the change in denoising with the change in $\tau_{1}=\zeta_{1}\sum_{j=1}^{n^{{}^{\prime }}}\frac{e_{j}}{n^{{}^{\prime }}}$, a randomly selected noisy MR image (2% noise) is denoised with varying values of $\zeta_{1}$. The obtained PSNR values for denoised images are plotted against different values for $\zeta_{1}$ in Figure 8. It can be observed that there is a slight increase in PSNR values for smaller values of $\zeta_{1}$. The increase in PSNR value at $\zeta_{1}=0.7$ compared to the image denoised with $\zeta_{1}=0.25$ can be explained by further reduction in noise at $\zeta_{1}=0.7$, as shown in Figure 8. The PSNR value is reduced significantly as the value for $\zeta_{1}$ is further increased. This can be explained by the corresponding image at $\zeta_{1}=1$, i.e., $\tau_{1}=1\sum_{j=1}^{n^{{}^{\prime }}}\frac{e_{j}}{n^{{}^{\prime }}}$, where even the textured/edge patches are considered as smooth and are denoised accordingly. This lead to huge image detail loss and thus the PSNR value drops. Hence, $\zeta_{1}=0.7$ is used for the experiments in this work. Similarly, the value of $\zeta_{2}$ is used to govern the minimum loss of image details while suppressing the noise and it has been observed that the value of 1.8 gives the best results.

### 4.2. Super Resolution of MR Image Volumes

**Figure 9:**
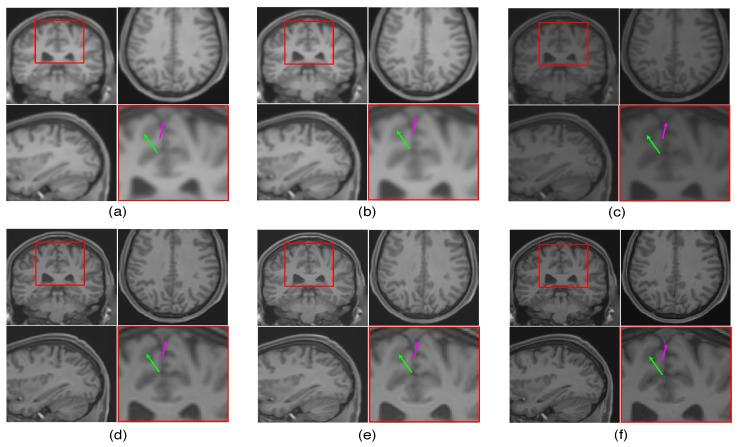
Illustration of reconstruction results for real MR image volume for super-resolution factor 2 using different super-resolution algorithms: (**a**) nearest neighbor interpolation, (**b**) spline interpolation, (**c**) non-local means [13], (**d**) low rank and total variation (LRTV) [12], (**e**) the proposed method, and (**f**) the original denoised high resolution (HR) image. Zoomed version of the red box shown in the axial slice is shown to demonstrate the difference (specifically in tissue boundaries indicated with arrows).

**Figure 10:**
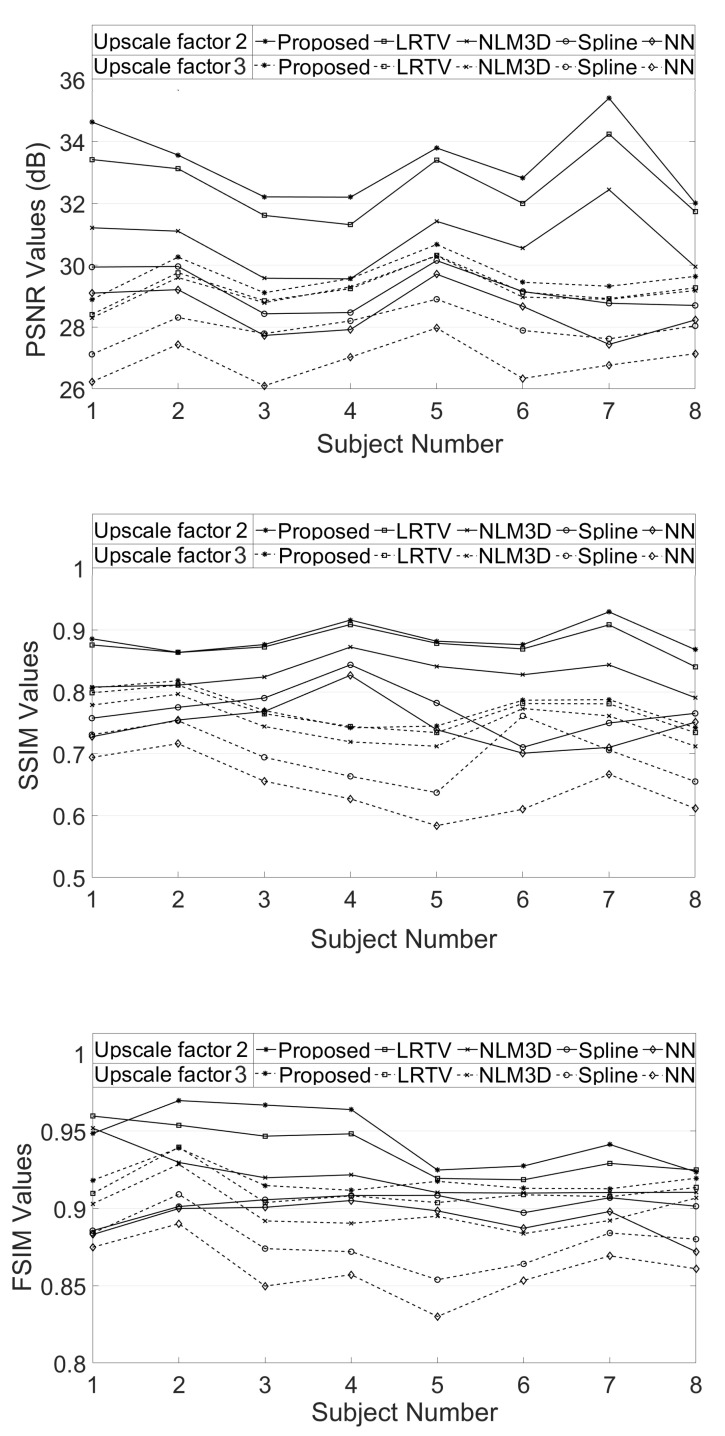
Demonstration of PSNR, structural similarity index (SSIM) and feature similarity index metric (FSIM) values obtained for super resolution of eight MR image volumes by upscale factors 2 and 3.

The LR image volumes are simulated by blurring the HR image volumes using a Gaussian kernel of the standard deviation of one voxel size and followed by downsampling. One MR image volume is randomly selected from dataset-II and super-resolved with super-resolution factor (SRF) 2 using the proposed SR approach and is compared with the existing state-of-the-art unsupervised methods NLM3D [13] and LRTV [12]. The reconstructed images using these approaches are shown in Figure 9. The highlighted region in the coronal slice is zoomed and shown in the red box. The pink and green arrows show the location for tissue boundary between cerebrospinal fluid (CSF) and gray matter, and white matter and gray matter, respectively. It can be observed that the proposed approach reconstructs the image details with relatively sharper tissue boundaries as well as better preservation of image details (near green arrow) as compared to the existing methods. Table 2 shows the mean PSNR, SSIM and FSIM values computed for different subjects for SRFs 2 and 3 reconstructed by different methods. The graphical representation of Table 2 is shown in Figure 10. It can be observed that the proposed approach provides comparably higher PSNR, SSIM, and FSIM values than existing methods, and thus illustrates the advantages of the proposed work in pixel-intensity, and structural and gradient-feature-based similarities, respectively.

A popular supervised approach in neural network super-resolution convolutional neural network (SR-CNN) [9] (after training using paired example LR-HR images) is also tested for SR of the MR image volumes used for evaluation of performance of proposed approach. It provides the maximum SSIM value 0.9012 and the average SSIM value reaches 0.8666 for (SRF = 2) which is comparable to the proposed approach.

**Figure 11:**
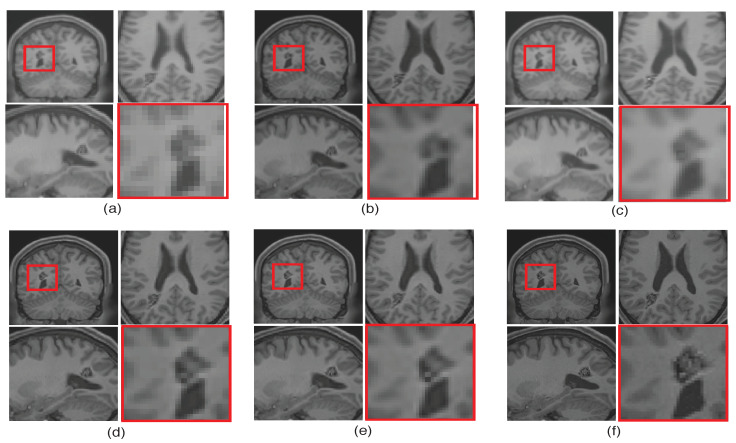
Illustration of super resolution results for structural deformity cavernoma in real MR images, by different algorithms: (**a**) nearest neighbor, (**b**) spline interpolation, (**c**) non local means in three dimensions (NLM3D) [13], (**d**) low rank total variation based method (LRTV) [12], (**e**) the proposed approach, and (**f**) the original HR image. Each slice in axial, sagittal, and coronal planes is shown. The zoomed version of the cavernoma region from the coronal slice is highlighted in red rectangle. Please zoom for better visualization.

To emphasize the significance of reconstruction of uncommon image details in the proposed SR method, we chose a real MR image volume with a cavernoma and improved its resolution by factor 2, as shown in Figure 11. It can be observed that the LRTV [12] approach in Figure 11d reduces the blur as compared to interpolation and NLM3D [13] in Figure 11b,c, but tends to produce staircase effect. It can be observed that the tissue boundaries and image details inside the cavernoma, shown in the red box, are more clear and distinct for the proposed method. In addition, the skull outlines can be seen to be well defined (in the sagittal plane) in the case of the proposed algorithm (see Figure 11e) as compared to existing methods.

#### Parameter Analysis for Optimal Selection of λ in the Proposed Super Resolution Approach

**Figure 12:**
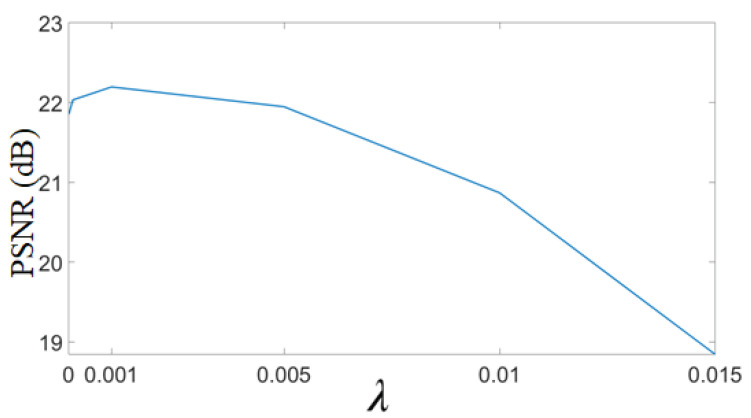
Optimal selection of parameter λ in the super resolution approach. A random MR image is selected and upscaled by factor 2 using the proposed super-resolution (SR) approach. The HR image is obtained with different values of λ in Equation (3), and the obtained PSNR values are plotted.

In the proposed approach, the weight to GPS-based regularizer is denoted by λ. It is a crucial parameter that decides the balance between the data fidelity term and the regularizer for gradient profiles. The optimal value of λ is chosen empirically based on the maximization of PSNR for estimated HR images. For this, an example MR image is upscaled by factor 2 with different values of λ, and PSNR values are plotted in Figure 12. It can be observed that with the high value of λ, i.e., beyond 0.005, the PSNR values dropped. Similarly for lesser values of λ, the PSNR value is less. The optimal of λ is thus chosen as 0.001 for which the PSNR value obtained is maximum, and holds true for another ensemble of MR images.

### 4.3. Resolution Improvement of Noisy MR Image Volumes

**Figure 13:**
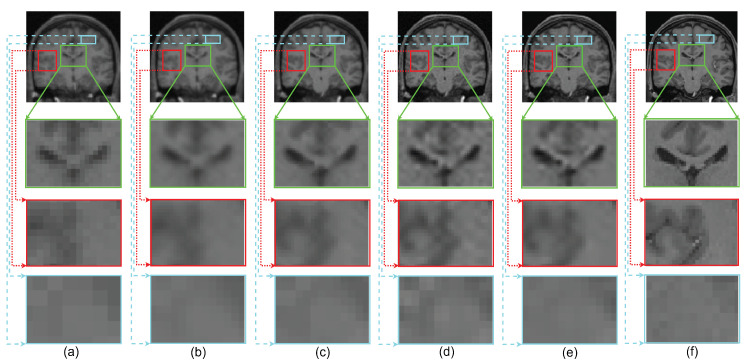
Demonstration of reconstruction quality of different kinds of regions/patches, region with edges (green boxes), region with texture (red boxes), and smooth region (cyan boxes), after super resolving real MR images degraded with downsampling factor 2 and 2% noise, using different algorithms. (**a**) NN interpolation of noisy LR image, (**b**) spline interpolation of denoised LR image, (**c**) NLM3D [13] applied on denoised LR image, (**d**) LRTV [12] applied on denoised LR images, (**e**) the proposed work applied on a noisy LR image, and (**f**) the original noise-free HR image.

The performance of the proposed single framework for the super resolution of noisy MR images has been evaluated here for 2% Gaussian noise and different upsampling factors. For existing SR methods, the images are first denoised using AONLM [40]. The qualitative results using different algorithms for upsampling a randomly selected noisy image by factor 2 are shown in Figure 13. To illustrate the significance of the proposed approach for efficient denoising as well as the preservation of image details, different kinds of regions are zoomed and shown separately. The green box in Figure 13 comprises the region with strong edges, the red box shows the region with minor edges and texture, and the cyan box shows the smooth region. It can be observed that the reconstructed image using spline interpolation and the non local means in three dimensions (NLM3D) approach in Figure 13b,c blur the image details in every kind of region and hence provide poorer contrast but with better denoising in each region. The LRTV method, however, tends to provide better contrast among tissues and preservation of image details, yet it is not able to provide efficiently denoised smooth regions (see red and cyan boxes in Figure 13d). The proposed work, on the contrary, can be observed to reconstruct strong edges while preserving the image details with limited noise (see green box Figure 13e), it can relatively better suppress the noise in patches with smooth and texture/minor edges as compared to LRTV [12] (can be seen in red and cyan boxes), and it can provide improved contrast as compared to NLM3D [13]. It demonstrates that the proposed method tends to improve the trade-off between the preservation of image details and suppression of noise. The obtained PSNR, SSIM, and FSIM values for different subjects are summarized in Table 3 and show that the proposed work performs relatively better than existing unsupervised methods when combined with supervised denoising method.

**Figure 14:**
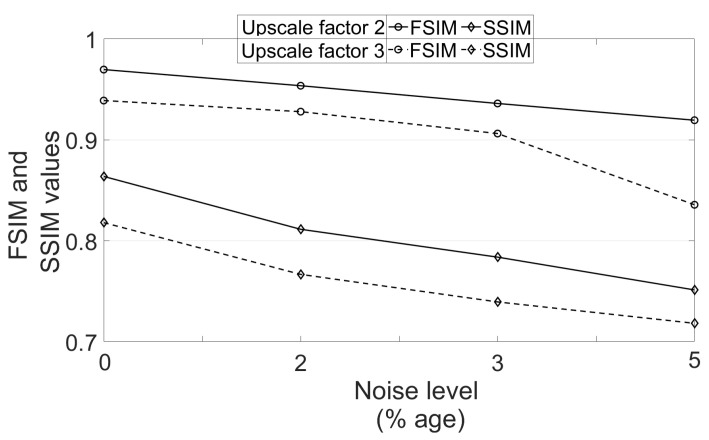
Analysis of FSIM and SSIM values for different noise variances and upscale factors.

To see the effect of different noise levels, a volume is randomly selected followed by degradation with downsampling factor 2 and varying noise levels (1% to 4%). The obtained PSNR values using different algorithms are mentioned in Table 4. It can be observed that the increase in PSNR values of images reconstructed by the proposed work are decreased as the noise level is increased and this is expected because restoring the image details for a high level of noise is a challenging problem. In addition, the PSNR metric represents an average measure of similarity and thus can be higher even for the blurred image as compared to the image with left out noise. This is observed to be true in case of 4% noise, where NLM3D [13] provides the blurred image with comparable PSNR. The FSIM and SSIM values are obtained for the estimated HR image using the proposed approach for varying variance of noise and upscaling factors, and are plotted in Figure 14. The metrics are obtained for one randomly chosen real MR image volume from dataset-II. It can be observed that the FSIM values decrease at a lesser rate when strength of noise is increased as compared to the case where the upscale factor is increased.

#### Analysis for Alzheimer Subjects

**Figure 15:**
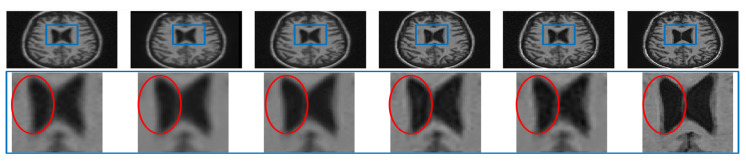
Illustration of super resolving the real MR images of an Alzheimer’s subject degraded with downsampling factor 2 and 2% noise. From Left: Interpolated noisy LR image, spline interpolation of denoised image, NLM3D-based super-resolved denoised image, LRTV-based super resolution of the denoised image, the proposed approach super resolving the noisy LR image, and the original noiseless HR image.

The clinical applications of the proposed approach can be valid only if no artifacts are introduced while denoising or super resolving. To experimentally verify this, a noisy LR image of an Alzheimer’s subject is restored by different algorithms and compared in the first row of Figure 15. The zoomed version of the region in the blue rectangle (highlighted in the first row) is shown in the second row. It can be observed that interpolated images and NLM3D [13] provide blurred image details. The LRTV [12] approach highlights the CSF flow artifact significantly (which is not as present in the noise-free HR image), may compromise the detection of pathology in the ventricular system, thus compromising the diagnostic capability. The image reconstructed by the proposed approach looks relatively noisy but clearer than existing methods and does not highlight any such artifact; hence, it can aid in accurate diagnosis. The same values of $\zeta_{1}=0.7$, $\zeta_{2}=1.8$, and λ=0.001 were used to process the MR images of an Alzheimer’s subject obtained from dataset-III as for processing dataset-II and dataset-I. It indicates the generalization of the proposed approach that uses the same values of parameters for different datasets.

## 5. Summary

This paper addresses the embedded framework for the super resolution of the noisy MR images. The smooth and textured/edge patches are categorized, without the prior/estimated knowledge of noise, and are denoised using different denoising strategies for efficient denoising. Further, the degradation of gradient profiles is addressed specifically for MR images to regularize the estimation of the HR image with clear tissue boundaries. The experimental section demonstrates the significance of proposed different denoising strategies for categorized patches to achieve efficient denoising. The importance of GPS constraint is demonstrated in the results section by clear tissue boundaries, i.e., reduced blur and reduced staircase effect, and higher FSIM values in estimated HR images. The experimental section demonstrates that the proposed unsupervised denoising approach performs comparably to the conventional [34] and a few recent supervised denoising approaches [25,35,40], which require prior knowledge of noise. The proposed integrated framework performs better than the existing unsupervised approaches [12,13] for super resolution of real MR images (with and without synthetically added noise). The proposed unsupervised SR method performs better than existing unsupervised methods for each kind of region (smooth/textured/strong edges) in noisy MR images, and thus improves the trade-off between denoising and smearing of image details. In addition, the proposed work performs comparable to the supervised deep learning-based SR approach [9] indicating the potential of unsupervised approaches.

## Figures and Tables

**Table 1:** Quantitative comparison (peak signal to noise ratio (PSNR) and structural similarity index (SSIM) values) of the proposed denoising approach with existing approaches. Each row indicates a different method and each column indicates a different noise level (in %, i.e., 2% means noise variance is 255×0.02=5.1 for image with 255 maximum intensity value) with mentioned distribution (Rician/Gaussian) and mentioned datasets (real/simulated dataset).

I/P				Rician Noise										Gaussian Noise									
	Method	Assumed Noise	Need Var.	Simulated Dataset					Real Dataset					Simulated Dataset					Real Dataset				
				3%	5%	7%	9%	11%	3%	5%	7%	9%	11%	3%	5%	7%	9%	11%	3%	5%	7%	9%	11%
**2D Image based Methods**	**Noisy** **Image**	-	-	30.41	26.00	23.12	20.98	19.28	20.45	19.87	19.14	18.32	17.47	30.39	25.95	23.03	20.85	19.10	20.38	19.70	18.84	17.91	16.96
				0.8401	0.7623	0.6946	0.6369	0.5874	0.5003	0.4914	0.4828	0.4747	0.4669	0.8397	0.7598	0.6886	0.6271	0.5746	0.4984	0.4862	0.4730	0.4600	0.4476
	**NLM**	Rice	Yes	35.88	32.62	30.23	28.37	26.74	34.54	31.45	29.06	27.18	25.64	35.87	32.66	30.36	28.72	27.48	34.66	31.88	29.93	28.55	27.55
				0.9239	0.8866	0.8469	0.8082	0.7694	0.8927	0.8301	0.7705	0.7204	0.6804	0.9237	0.8857	0.8445	0.8073	0.7763	0.8942	0.8372	0.7875	0.7489	0.7192
	**UNLM**	Rice	Yes	36.35	33.21	30.90	29.13	27.77	34.44	31.67	29.78	28.42	27.38	36.26	32.94	30.30	28.10	26.17	34.49	31.66	29.46	27.46	25.50
				0.9307	0.8990	0.8639	0.8296	0.7984	0.8955	0.8499	0.8018	0.7569	0.7191	0.9304	0.8966	0.8522	0.8020	0.7494	0.8967	0.8537	0.8083	0.7638	0.7197
	**VST** **BM3D**	Rice	Yes	36.28	33.54	31.75	30.36	29.24	35.19	32.87	31.33	30.07	28.90	36.12	32.91	30.28	27.93	25.86	35.08	32.16	29.31	26.49	24.06
				0.9304	0.9070	0.8858	0.8635	0.8423	0.9018	0.8701	0.8409	0.8112	0.7810	0.9297	0.8999	0.8607	0.8136	0.7634	0.9024	0.8701	0.8334	0.7861	0.7317
	**Proposed**	**Nill**	**No**	**34.21**	**32.58**	**30.19**	**28.21**	**25.80**	**34.57**	**32.04**	**29.08**	**27.08**	**25.66**	**33.86**	**32.14**	**30.14**	**28.26**	**25.93**	**34.10**	**32.03**	**29.74**	**26.80**	**24.08**
				**0.9235**	**0.9010**	**0.8564**	**0.8045**	**0.7623**	**0.8903**	**0.8560**	**0.7989**	**0.7247**	**0.6862**	**0.9192**	**0.8953**	**0.8585**	**0.8182**	**0.7559**	**0.8828**	**0.8593**	**0.8107**	**0.7350**	**0.5677**
**3D Volume based Methods**	**ORNLM**	Rice	Yes	36.34	33.45	31.53	29.74	27.63	35.13	32.31	30.53	29.20	28.28	36.00	33.03	31.37	30.23	29.35	35.54	33.15	31.55	30.29	29.32
				0.9312	0.9067	0.8819	0.8492	0.8016	0.9130	0.8719	0.8400	0.8132	0.7902	0.9308	0.9051	0.8843	0.8664	0.8506	0.9164	0.8821	0.8536	0.8295	0.8080
	**AONLM**	Rice	Yes	36.28	33.64	31.95	30.61	29.48	34.74	32.71	31.12	29.95	28.77	36.10	33.43	31.74	30.47	29.44	34.67	32.68	31.27	30.16	29.24
				0.9295	0.9056	0.8842	0.8628	0.8421	0.9061	0.8794	0.8498	0.8242	0.7964	0.9293	0.9041	0.8822	0.8615	0.8422	0.9044	0.8780	0.8534	0.8304	0.8090
	**MRNLM**	Rice	Yes	36.05	33.26	31.38	29.64	27.59	35.49	32.84	31.00	29.58	28.60	35.47	32.57	30.96	29.86	29.00	35.52	33.05	31.36	29.98	28.95
				0.9329	0.9091	0.8852	0.8543	0.8087	0.9160	0.8795	0.8484	0.8212	0.7991	0.9314	0.9050	0.8837	0.8655	0.8494	0.9162	0.8822	0.8531	0.8274	0.8057
	**ODCT**	Rice	Yes	35.88	32.69	30.35	28.26	26.13	31.20	28.96	27.47	27.94	27.78	35.87	32.46	29.94	28.05	26.59	32.12	30.83	30.28	28.50	27.18
				0.9295	0.9017	0.8694	0.8289	0.7754	0.8575	0.8103	0.7779	0.7861	0.7765	0.9303	0.9001	0.8654	0.8327	0.8019	0.8742	0.8547	0.8411	0.7932	0.7496
	**PRINLM**	Rice	Yes	36.26	32.87	30.29	28.11	25.79	30.93	27.34	26.83	27.64	27.18	36.06	32.28	29.51	27.55	26.03	31.70	30.87	30.13	28.00	26.61
				0.9373	0.9106	0.8757	0.8281	0.7616	0.8533	0.8091	0.7729	0.7956	0.7714	0.9363	0.9035	0.8634	0.8229	0.7793	0.8677	0.8594	0.8372	0.7751	0.7129
	**VST** **BM4D**	Rice	Yes	36.65	33.90	32.16	30.80	29.71	35.48	33.14	31.54	30.25	29.29	36.57	33.42	30.75	28.35	26.15	35.43	32.54	29.63	26.72	24.21
				0.9353	0.9142	0.8945	0.8746	0.8555	0.9057	0.8765	0.8496	0.8227	0.7985	0.9358	0.9089	0.8715	0.8271	0.7777	0.9067	0.8772	0.8409	0.7936	0.7400
	**VST** **NLM3D**	Rice	Yes	36.04	33.21	31.34	29.88	28.62	35.00	32.57	30.91	29.57	28.42	35.93	32.65	29.96	27.52	25.29	34.86	31.89	28.93	25.91	23.49
				0.9278	0.9007	0.8754	0.8508	0.8252	0.9061	0.8730	0.8424	0.8130	0.7838	0.9272	0.8929	0.8510	0.8024	0.7485	0.9066	0.8720	0.8296	0.7731	0.7146

Here, red color denotes the highest PSNR/SSIM values obtained, bold numbers denote the PSNR and SSIM values obtained using the proposed work. Blue color denotes the PSNR/SSIM values comparable (within 0.5 dB PSNR range) to the proposed work obtained using different approaches.

**Table 2:** PSNR, SSIM, and FSIM values obtained using different algorithms for super resolution of real MR images in all three directions by factors 2 and 3.

	Subject	Metric	NN	Spline	NLM3D	LRTV	Proposed
SRF = 2	1	PSNR	29.0981	29.9406	31.2103	33.4160	**34.63**
		SSIM	0.7275	0.7575	0.8079	0.8759	**0.8859**
		FSIM	0.8831	0.8856	0.9519	0.9597	**0.9484**
	2	PSNR	29.21	29.96	31.10	33.12	**33.56**
		SSIM	0.7546	0.7750	0.8109	0.8637	**0.8638**
		FSIM	0.8999	0.9012	0.9297	0.9538	**0.9697**
	3	PSNR	27.73	28.43	29.58	31.61	**32.21**
		SSIM	0.7682	0.7900	0.8241	0.8727	**0.8765**
		FSIM	0.9007	0.9056	0.9198	0.9467	**0.9668**
	4	PSNR	27.92	28.47	29.56	31.31	**32.20**
		SSIM	0.8269	0.8437	0.8724	0.9089	**0.9161**
		FSIM	0.9051	0.9083	0.9217	0.9482	**0.9639**
	5	PSNR	29.71	30.15	31.42	33.40	**33.79**
		SSIM	0.7389	0.7821	0.8413	0.8786	**0.8819**
		FSIM	0.8984	0.9084	0.9102	0.9194	**0.9248**
	6	PSNR	28.67	29.15	30.55	32.00	**32.82**
		SSIM	0.7009	0.7104	0.8277	0.8695	**0.8762**
		FSIM	0.8872	0.8972	0.9099	0.9185	**0.9274**
	7	PSNR	27.44	28.77	32.44	34.24	**35.40**
		SSIM	0.7103	0.7497	0.8436	0.9083	**0.9293**
		FSIM	0.8980	0.9069	0.9103	0.9291	**0.9413**
	8	PSNR	28.23	28.70	29.95	31.73	**32.01**
		SSIM	0.7511	0.7653	0.7907	0.8407	**0.8683**
		FSIM	0.8720	0.9014	0.9104	0.9248	**0.9236**
SRF = 3	1	PSNR	26.23	27.12	28.30	28.41	**28.89**
		SSIM	0.6941	0.7308	0.7787	0.7986	**0.8066**
		FSIM	0.8749	0.8840	0.9030	0.9096	**0.9182**
	2	PSNR	27.44	28.31	29.60	29.76	**30.26**
		SSIM	0.7163	0.7534	0.7963	0.8108	**0.8180**
		FSIM	0.8901	0.9091	0.9285	0.9396	**0.9390**
	3	PSNR	26.10	27.79	28.79	28.85	**29.11**
		SSIM	0.6557	0.6943	0.7441	0.7647	**0.7699**
		FSIM	0.8498	0.8740	0.8919	0.9038	**0.9148**
	4	PSNR	27.03	28.20	29.30	29.24	**29.57**
		SSIM	0.6271	0.6635	0.7192	0.7442	**0.7419**
		FSIM	0.8571	0.8720	0.8904	0.9081	**0.9117**
	5	PSNR	27.97	28.90	30.30	30.32	**30.67**
		SSIM	0.5838	0.6372	0.7121	0.7342	**0.7448**
		FSIM	0.8301	0.8539	0.8949	0.9038	**0.9175**
	6	PSNR	26.34	27.89	28.97	29.13	**29.45**
		SSIM	0.6103	0.7612	0.7731	0.7812	**0.7865**
		FSIM	0.8534	0.8641	0.8837	0.9088	**0.9130**
	7	PSNR	26.77	27.62	28.90	28.92	**29.32**
		SSIM	0.6668	0.7059	0.7612	0.7807	**0.7875**
		FSIM	0.8693	0.8841	0.8922	0.9076	**0.9127**
	8	PSNR	27.14	28.04	29.18	29.27	**29.64**
		SSIM	0.6115	0.6551	0.7121	0.7341	**0.7421**
		FSIM	0.8610	0.8801	0.9067	0.9135	**0.9195**

Bold numbers indicate the best performance of respective metrics.

**Table 3:** Quantitative results for different algorithms for super resolution of real MR images with upscaling factors 2 and 3, degraded with synthetically added 2% noise.

	Subjects	Metric	NN	Spline	NML3D	LRTV	Proposed
SRF = 2	1	PSNR	29.01	29.50	30.17	31.29	**31.95**
		SSIM	0.6893	0.7099	0.7562	0.7972	**0.7983**
		FSIM	0.8820	0.8841	0.9102	0.9404	**0.9546**
	2	PSNR	29.47	30.25	30.97	32.20	**32.81**
		SSIM	0.7234	0.7802	0.7971	0.8068	**0.8114**
		FSIM	0.8987	0.8981	0.9178	0.9456	**0.9537**
	3	PSNR	27.98	28.70	29.44	30.61	**31.26**
		SSIM	0.7012	0.7289	0.7313	0.8029	**0.8032**
		FSIM	0.8979	0.8986	0.9186	0.9443	**0.9530**
	4	PSNR	31.64	32.96	33.59	34.64	**34.52**
		SSIM	0.7901	0.8001	0.8162	0.8512	**0.8524**
		FSIM	0.9020	0.9028	0.9198	0.9418	**0.9507**
	5	PSNR	28.31	29.00	29.68	30.66	**31.23**
		SSIM	0.7173	0.7214	0.7445	0.7991	**0.8041**
		FSIM	0.8904	0.9008	0.9038	0.9048	**0.9121**
	6	PSNR	29.13	29.54	30.26	31.43	**31.97**
		SSIM	0.7529	0.7712	0.7830	0.8090	**0.8095**
		FSIM	0.8694	0.9027	0.9071	0.9103	**0.9174**
	7	PSNR	29.21	29.80	30.49	31.54	**32.32**
		SSIM	0.7374	0.7442	0.7834	0.8186	**0.8291**
		FSIM	0.8943	0.9041	0.9094	0.9194	**0.9255**
	8	PSNR	28.47	29.12	29.83	30.86	**31.47**
		SSIM	0.7404	0.7619	0.7910	0.8138	**0.8108**
		FSIM	0.8632	0.8926	0.9088	0.9122	**0.9198**
SRF = 3	1	PSNR	26.45	26.97	27.87	27.62	**28.08**
		SSIM	0.7192	0.7231	0.7450	0.7401	**0.7532**
		FSIM	0.8611	0.8814	0.9006	0.9059	**0.9134**
	2	PSNR	27.19	27.86	29.17	28.99	**29.26**
		SSIM	0.7267	0.7342	0.7662	0.7580	**0.7667**
		FSIM	0.8854	0.9072	0.9223	0.9232	**0.9280**
	3	PSNR	26.97	27.24	28.48	28.28	**28.49**
		SSIM	0.7142	0.7208	0.7221	0.7241	**0.7354**
		FSIM	0.8443	0.8654	0.8907	0.9001	**0.9062**
	4	PSNR	26.95	27.35	28.72	28.29	**28.93**
		SSIM	0.6707	0.6744	0.6819	0.6804	**0.6880**
		FSIM	0.8486	0.8662	0.8888	0.9000	**0.9026**
	5	PSNR	28.16	28.88	29.52	28.93	**29.44**
		SSIM	0.6423	0.6487	0.6560	0.6446	**0.6689**
		FSIM	0.8218	0.8501	0.8845	0.8875	**0.8974**
	6	PSNR	26.91	27.12	28.33	27.90	**28.43**
		SSIM	0.7128	0.7146	0.7257	0.7225	**0.7351**
		FSIM	0.8424	0.8631	0.8734	0.9075	**0.9047**
	7	PSNR	27.19	27.57	28.62	28.29	**28.83**
		SSIM	0.6367	0.6456	0.6701	0.6647	**0.6807**
		FSIM	0.8534	0.8732	0.8982	0.9041	**0.9105**
	8	PSNR	27.05	27.67	28.49	29.09	**29.63**
		SSIM	0.6848	0.6896	0.7015	0.6971	**0.7035**
		FSIM	0.8517	0.8760	0.9028	0.9064	**0.9142**

Bold numbers indicate the best performance of respective metrics.

**Table 4:** PSNR values for reconstructed images of randomly selected subject degraded with downsampling factor 2 and different levels of noise.

Noise Level	Interpolation	NLM3D [13]	LRTV [12]	Proposed
1%	30.12	30.5	31.67	32.19
2%	30.26	30.58	31.43	31.78
3%	29.75	30.00	30.34	30.45
4%	29.54	29.62	29.28	29.34

## Data Availability

Not applicable.
